# Live and let die: signaling AKTivation and UPRegulation dynamics in SARS-CoVs infection and cancer

**DOI:** 10.1038/s41419-022-05250-5

**Published:** 2022-10-03

**Authors:** Mariana Suaya, Gonzalo Manuel Sánchez, Antonella Vila, Analía Amante, María Cotarelo, Mercedes García Carrillo, Matías Blaustein

**Affiliations:** 1 Departamento de Fisiología, Biología Molecular y Celular, Facultad de Ciencias Exactas y Naturales (FCEN), Universidad de Buenos Aires (UBA), Buenos Aires, Argentina; 2 Instituto de Biociencias, Biotecnología y Biología Traslacional (iB3), FCEN, UBA, Buenos Aires, Argentina; 3 Consejo Nacional de Investigaciones Científicas y Técnicas (CONICET), Buenos Aires, Argentina

**Keywords:** Mechanisms of disease, Cancer, Infection, Cell signalling

## Abstract

The severe acute respiratory syndrome coronavirus 2 (SARS-CoV-2) is the pathogen responsible for the coronavirus disease 2019 (COVID-19) pandemic. Of particular interest for this topic are the signaling cascades that regulate cell survival and death, two opposite cell programs whose control is hijacked by viral infections. The AKT and the Unfolded Protein Response (UPR) pathways, which maintain cell homeostasis by regulating these two programs, have been shown to be deregulated during SARS-CoVs infection as well as in the development of cancer, one of the most important comorbidities in relation to COVID-19. Recent evidence revealed two way crosstalk mechanisms between the AKT and the UPR pathways, suggesting that they might constitute a unified homeostatic control system. Here, we review the role of the AKT and UPR pathways and their interaction in relation to SARS-CoV-2 infection as well as in tumor onset and progression. Feedback regulation between AKT and UPR pathways emerges as a master control mechanism of cell decision making in terms of survival or death and therefore represents a key potential target for developing treatments for both viral infection and cancer. In particular, drug repositioning, the investigation of existing drugs for new therapeutic purposes, could significantly reduce time and costs compared to *de novo* drug discovery.

## Facts

- The AKT and UPR pathways maintain cell homeostasis, regulating cell decision making mechanisms in terms of cell survival and death.
- The AKT and UPR pathways are co-opted both during viral infection and cancer development.
- The AKT and UPR pathways constitute a unified homeostatic control system, emerging as a master control mechanism both in physiological and pathological conditions.
- The crosstalk between the AKT and UPR pathways represents a key potential target in the development of treatments for both viral infection and cancer.

## Introduction: COVID-19, cancer and therapeutic targets

The coronavirus disease 2019 (COVID-19) outbreak was first reported in December 2019 in Wuhan, China [1]. Its causative agent -the severe acute respiratory syndrome coronavirus 2 (SARS-CoV-2)- is an enveloped positive-sense RNA virus [2], belonging to the lineage B (Sarbecovirus) of the Betacoronavirus family. The genomic sequence of SARS-CoV-2 has 29,903 nucleotides in length [3], and it is closely related to SARS-CoV (79% genetic similarity) [4]. It presents 10–12 putative open reading frames (ORFs), including four specifically coding for its structural proteins: Spike (S), Envelope (E), Membrane (M), and Nucleocapsid (N). The Spike protein drives SARS-CoV-2 tropism and mediates cellular infection by binding to the angiotensin-converting enzyme (ACE-2) [5]. Since its first report in 2019, SARS-CoV-2 has rapidly spread worldwide, resulting in hundreds of millions of people infected and over 6 million deaths.

SARS-CoV-2 lethality is increased in people with different comorbidities, of which cancer, a group of diverse pathologies characterized by uncontrolled cell proliferation and dissemination, is one of the most prominent [6]. This has been related to the fact that many antitumor therapies generate immunosuppression [7]. Cancer patients are not only frequently immunosuppressed, but also suffer other side effects from antitumor treatments, having a higher risk of contracting infections, developing complications, and requiring intensive care, which is why they were considered a population at risk during the pandemic [8]. As an example, it has been reported that having received chemotherapy within 4 weeks prior to the onset of COVID-19 symptoms is associated with an increased risk of mortality [7, 8], indicating that the quality of the immune system can be a determining factor in the severity of the disease. Since there is evidence that antitumor treatments that compromise the immune system correlate with worse prognosis in COVID-19 patients, the development of alternative therapies that do not affect the immune system for cancer patients in the current context of pandemic is of great importance. In this sense, seeking pharmacological therapies associated with human proteins, which have a lower mutation rate than viral proteins, could be an effective strategy, complementary to the use of vaccines, which may present variability in the efficacy against different emerging variants of the virus. Furthermore, such a strategy could be relevant in the potential case of a future epidemic or pandemic caused by another strain or type of coronavirus. Of particular interest for this purpose are the AKT [9] and the Unfolded Protein Response (UPR) [10] pathways, which maintain cell homeostasis by regulating cell survival and cell death, two opposite cell programs whose control is hijacked by viral infections. As it will be reviewed below, these pathways have been shown to be deregulated during SARS-CoV infection as well as in the development of cancer. Feedback regulation between AKT and UPR pathways emerges as a master control mechanism of cell decision making in terms of survival or death and therefore represents a key potential target for developing treatments for cancer and viral infection (in particular, COVID-19). The repositioning of drugs, a strategy whereby new indications are found for existing drugs, could significantly reduce time and costs compared to de novo drug discovery [11].

### AKT, cancer and disease

AKT (also known as protein kinase B or PKB) is a serine/threonine kinase member of the AGC family of protein kinases. In mammals, three cellular homologs of AKT (AKT1, AKT2, and AKT3) have been found, each transcribed from separate genes [12–14].

AKT contains an N-terminal pleckstrin homology (PH) domain that interacts with phosphatidylinositol (3,4,5)-triphosphate (PIP3), a central kinase (CAT) domain, and a C-terminal extension (EXT) that contains a hydrophobic motif (HM) with homology to other AGC kinases [15]. AKT plays a central role in growth, proliferation, differentiation, glucose uptake, metabolism, angiogenesis, protein translation, cell survival and apoptosis, and constitutes a vastly studied model of how signaling proteins transduce and process extracellular information into cell decisions and fates [9, 16].

**Fig. 1:**
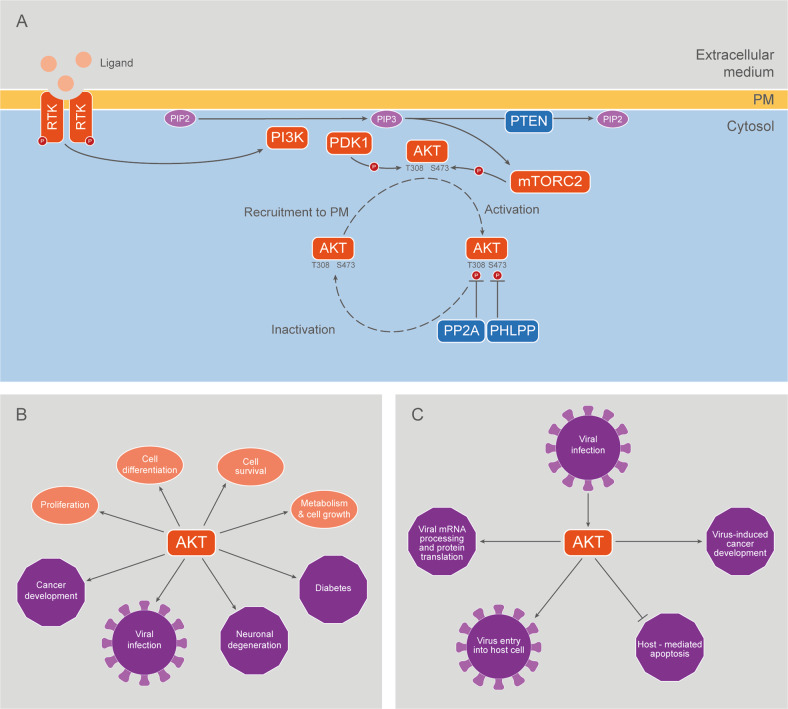
The AKT pathway. **A** A traditional perspective of AKT activation assumes that PI3K, which converts PM-associated PIP2 to PIP3 in response to Growth Factors (GF) and other stimuli, leads to PIP3-mediated recruitment of cytosolic AKT to PM. The conformational change elicited by AKT PH domain binding to PIP3 allows phosphorylation of AKT by PDK1 and mTORC2. As a result, activated AKT concertedly regulates a great variety of targets and functions in the cytosol and the nucleus, among other subcellular compartments. **B** Examples of physiological and pathological processes in which AKT is implicated. **C** Key steps of viral infection regulated by AKT.

Traditional activation of AKT occurs when cognate ligands such as growth factors bind to tyrosine kinase receptors, resulting in their autophosphorylation, a process followed by the recruitment of small G proteins such as Ras (Fig. 1). One Ras effector is class I phosphatidylinositol-3-kinase (PI3K), which converts plasma membrane (PM) associated phosphatidylinositol-4,5-biphosphate (PIP2) to PIP3. AKT is then recruited to the PM by binding to PIP3 through its PH domain. This binding induces a conformational change that allows phosphorylation of AKT1 in T308 (T309 and T305 in AKT2 and AKT3, respectively) by Phosphoinositide-dependent kinase-1 (PDK1) [17] and in S473 (S474 and S472 in AKT2 and AKT3, respectively) by the mammalian target of rapamycin complex 2 (mTORC2) [18], resulting in AKT activation (Fig. 1). On the other hand, dephosphorylation of AKT leads to the termination of AKT activation: protein phosphatase 2 A (PP2A) downregulates AKT activity by inducing dephosphorylation of T308 from AKT1, while PH domain leucine-rich repeat protein phosphatase (PHLPP) suppresses AKT activity by dephosphorylating AKT1 in S473 [19, 20]. Also, dephosphorylation of PIP3 to PIP2, induced by phosphatidylinositol 3,4,5-trisphosphate 3-phosphatase (PTEN), downregulates recruitment of AKT to PM and, consequently, results in reduced AKT activity [21] (Fig. 1).

In the last few years, novel phosphorylation sites have been reported at the carboxy-terminus of AKT, which contribute to its activation [22], maturation, folding and stability [17, 23]. Additionally, AKT can be phosphorylated in several tyrosine residues, which seems to be a prerequisite to both T308 and S473 phosphorylations [24, 25]. Interestingly, in addition to phosphorylation, other AKT post-translational modifications (PTMs) have been described: O-glycosylation, ubiquitination, acetylation, oxidation and SUMOylation [16, 26]. It was also demonstrated that AKT is proline-hydroxylated [27] (Guo et al., 2016), methylated [28] and palmitoylated [29].

In addition to PM, some studies show that AKT can be also found in internal membranes such as those of the endoplasmic reticulum (ER), mitochondria, lysosomes, Golgi and early endosomes [29–34]. Once activated, AKT acts over multiple targets located in the nucleus, cytosol and different internal cell membranes. AKT is known to phosphorylate a large and diverse group of proteins containing the AKT consensus motif (RXRXXS*/T*B), where “S*/T*” represents the phosphorylatable serine or threonine, X is any residue, and B is a bulky hydrophobic residue [9]. Given the importance of the AKT pathway in all the aforementioned processes, it is consistent that its dysregulation has been associated with a wide variety of human diseases, including pathological cardiac hypertrophy, diabetes, neurodegeneration, vascular disorders, different types of cancer and viral infections [35–38] (Fig. 1).

The role of AKT in cancer development has been supported by numerous animal tumor models [39]. AKT was also shown to regulate hormone independence and tumor differentiation [40]. Additionally, AKT1, AKT2, and AKT3 isoforms are found to be overexpressed in several human cancers [35]. These results highlight the critical role of the PI3K/AKT pathway in cancer development.

In this context, AKT represents an attractive therapeutic target for cancer treatment and, as discussed below, for the treatment of specific viral infections. Given the variety of functions carried out by this kinase, only a fraction of them are relevant to tumor and viral progression. Thus, the choice of drug targets must take into account the adverse effects that may result from the inhibition of cellular processes regulated by AKT, such as those derived from its participation in other cell signaling pathways. Therefore, a deep understanding of the molecular mechanisms underlying the regulation of the activity of this protein kinase acquires special relevance [41]. In particular, inhibitors of the AKT pathway have been developed for therapeutic treatments and some of them are beginning to be used in clinical trials [42, 43].

### UPR, cancer and disease

**Fig. 2:**
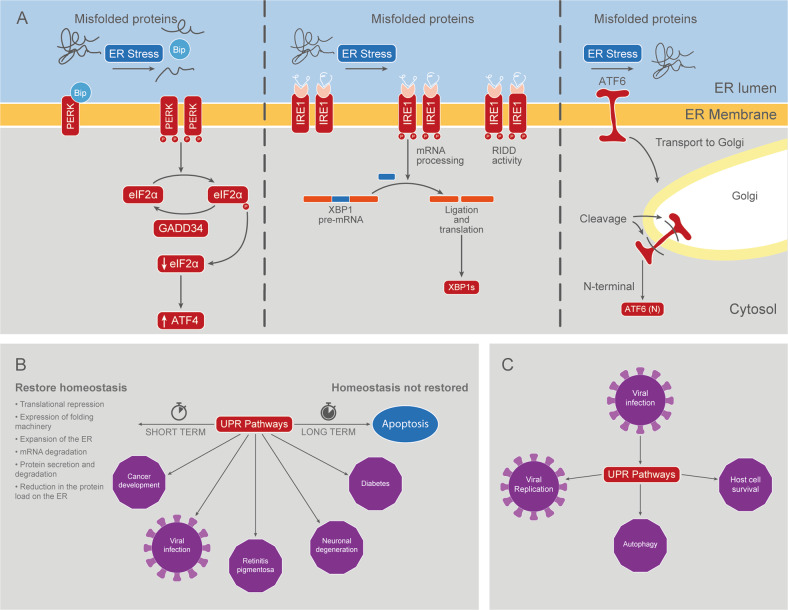
The UPR pathways. **A** Activation of the UPR pathways. Three ER stress transducers have been identified: IRE1, ATF6 and PERK. These integral membrane proteins sense the protein folding status in the ER lumen and communicate this information to cytosolic target proteins that translocate to the nucleus to modulate gene expression. **B** Examples of physiological and pathological processes in which the UPR is implicated. **C** Key steps of viral infection regulated by the UPR.

In eukaryotic cells, most of the secreted and transmembrane proteins fold and mature in the lumen of the endoplasmic reticulum (ER). The flow within this cell compartment is variable and can rapidly change in response to different contexts such as cell differentiation, diverse environmental conditions, and different physiological states of the cell. To handle this dynamic situation, cells developed a control system collectively known as the UPR, which is activated during different ER stress situations, such as the accumulation of misfolded proteins (Fig. 2).

The activation of the UPR mediates the expansion of ER membranes and the production of proteins that are part of the protein folding machinery and that reside in the ER lumen, such as chaperones and foldases. These processes help to increase the folding capacity of the cell. In parallel, this is accompanied by mechanisms that decrease the flow of proteins that transiently enter the ER. If the activity of the UPR persists, which means that cellular homeostasis cannot be restored, then the mechanisms mentioned above are turned off, and apoptosis is induced instead (Fig. 2). In this sense, the UPR consists of numerous feedback loops that establish and maintain the balance between survival and cell death [10].

Three different signaling UPR pathways have been described (Fig. 2), the main players of which are ER transmembrane proteins: inositol-requiring protein-1 (IRE1), activating transcription factor-6 (ATF6) and protein kinase RNA (PKR)-like ER kinase (PERK).

PERK is linked through its luminal domain to the binding immunoglobulin protein (BiP), also known as the glucose-regulated protein 78 (GRP78), and its activation occurs when BiP dissociates to bind misfolded proteins. This leads to oligomerization and transphosphorylation of PERK, thus activating its tyrosine kinase domain. Active PERK phosphorylates the eukaryotic translation initiation factor α (eIF2α), inactivating it, and inhibiting CAP-dependent messenger RNA (mRNA) translation initiation (Fig. 2). In this way, it favors the re-establishment of cellular homeostasis in the short run by reducing the load of synthesized proteins [44]. However, the reduction in active eIF2α levels favors the translation of activating transcription factor 4 (ATF4). Once induced, the ATF4 transcription factor activates a cluster of genes involved in the homeostatic response. ATF4 also regulates the transcription of the C/EBP homologous protein (CHOP) mRNA, and together with CHOP they induce the expression of the growth arrest and DNA damage-inducible protein (GADD34), which finally dephosphorylates eIF2α, allowing for global protein translation to resume [45]. Then, if UPR activation is maintained, apoptosis is promoted through CHOP, a transcription factor involved in cell arrest and apoptosis [46].

IRE1 is also linked through its luminal domain to BiP. After oligomerization and phosphorylation of IRE1, its cytosolic domain RNAse is activated, catalyzing an unconventional cytosolic “splicing” of X-Box Binding Protein 1 (XBP) 1mRNA [47, 48] (Fig. 2). In humans, splicing of the 26 nucleotide fragment produces a shift in the reading frame that encodes a version of XBP1 with a different amino acid sequence and higher molecular weight, known as spliced XBP1 or XBP1s [48]. XBP1s is a transcription factor that induces the expression of genes that contain an UPR response element (UPRE) or a hormone-specific response element (HRE) in their promoters, i.e. genes linked to folding, glycosylation, the endoplasmic-reticulum-associated protein degradation (ERAD) pathway, lipid biogenesis and/or vesicular trafficking [49]. IRE1 can also bind to and degrade other mRNAs through a process called IRE1-dependent regulated messenger degradation (RIDD) [50, 51]. While low levels of RIDD activity facilitate homeostasis by degrading ER-associated mRNAs, prolonged RIDD activity degrades mRNA coding for proteins that contribute to cell survival, thus promoting apoptosis (Fig. 2).

Finally, ATF6 is a transcription factor initially synthesized as an ER transmembrane protein. During UPR, ATF6 migrates to the Golgi apparatus via vesicular transport (Fig. 2). There, it is cleaved by site 1 and site 2 proteases (S1P and S2P) that sequentially remove its luminal and transmembrane domain, respectively, in a process called regulated intramembrane proteolysis (RIP) [52]. The N-terminal (cytosolic) domain migrates from the Golgi to the nucleus where it binds to UPRE regions and activates target genes that play a role in the recovery of ER homeostasis, especially genes that code for chaperones such as GRP78 and GRP94 (Fig. 2). Historically, the ATF6 pathway has been associated with pro-survival functions, but in recent years, evidence has emerged that ATF6 is a major inducer of CHOP [53].

In summary, UPR mainly induces translational silencing, transcriptional activation of molecular folding machinery, mRNA degradation, and stimulates both protein secretion and degradation [10, 54]. These processes promote cell survival in the short term, but if homeostasis is not restored, cell cycle arrest and apoptosis are promoted instead [10] (Fig. 2).

Dysregulation of UPR is reported in various diseases, such as cancer, diabetes, viral infection and neurodegenerative diseases [10, 55]. When homeostasis cannot be reestablished, UPR can function as a trigger for apoptosis in cells that are beneficial to the body. On the other hand, it can also have a cytoprotective effect by killing cells that are dangerous for the rest of the body [10]. Examples of the former are retinitis pigmentosa and other inherited forms of blindness, in which retinal cells undergo apoptosis when misfolded mutant rhodopsins are produced [56]. Examples of the latter are cancer and viral infections. Although in some types of cancer the UPR may have a protective effect for the host by triggering apoptosis in tumor cells [57], for the most part UPR functions as an adaptive survival mechanism [58], favoring tumor growth by allowing cancer cells to survive unfavorable conditions [59]. Furthermore, there is recent evidence indicating that, in addition to promoting tumor progression, activation of the UPR may also limit the effectiveness of chemotherapy, contributing to the development of chemoresistance [60]. It is in this context that the UPR has become an attractive therapeutic target for many types of cancer in recent years. In the case of viral infections, viruses exploit the UPR by increasing the ER’s ability to assist in viral replication, and in many cases, they make use of late apoptosis to be released in vesicles without being detected by the immune system [61] (Fig. 2).

### AKT, viral infection and SARS-CoV-2

#### AKT regulation during viral infection

Several viral proteins are capable of upregulating PI3K/AKT signaling by modulating this pathway at multiple points [38] (Supplementary Table 1 and Fig. 1). For example, the NS1 protein of Influenza A virus (IAV) binds to the p85β regulatory subunit of PI3K, increasing PI3K activity and resulting in the phosphorylation of AKT [62]. It has also been reported that NS1 directly interacts with phosphorylated AKT, enhancing AKT activity [63]. The ST protein of the simian virus 40 (SV40) binds to phosphatase PP2A, preventing AKT from being dephosphorylated and therefore inactivated [37, 64]. A similar mechanism has been proposed for the E7 protein of the Human Papillomavirus (HPV) [65].

Host organisms use apoptosis as an instrument to resist viral infections. Thus, activating the PI3K/AKT signaling pathway is a strategy used by some viruses to delay host cell apoptosis and extend viral replication, especially at the earliest stages of infection (Supplementary Table 1). As an example, NS1 and NS2 proteins of Respiratory Syncytial virus (RSV), which are expressed early in infection, induce the activation of NF-B and PI3K/AKT pathways [66]. Once AKT is activated, it phosphorylates MDM2 and leads to the degradation of p53, a pro-apoptotic protein [67]. Similarly, it has been shown that the activation of the PI3K/AKT pathway in cells infected with Sendai virus (SV) leads to the stabilization of XIAP, an anti-apoptotic protein that blocks caspase 9, preventing early apoptosis [68, 69]. Likewise, activation of the PI3K/AKT pathway in Newcastle Disease Virus (NDV) infected cells promotes an antiapoptotic response, as suggested by the fact that treatment with LY294002, a PI3K inhibitor, results in cleavage of PARP and caspase 3 at early stages of infection [70]. Vaccinia virus (VACV) and Cowpox virus (CPXV), two members of the *Poxviridae* family, also activate the PI3K/AKT pathway to avoid early apoptosis [71]. Furthermore, the PI3K/AKT pathway appears to play a key role in both VACV and CPVX mRNA expression, viral assembly and morphogenesis, which occurs at a late stage of viral infection [37, 38, 71, 72] (Supplementary Table 1).

It has also been reported that, in some cases, the AKT pathway has a role in early internalization events and endocytosis. Some viruses, like IAV, Hepatitis C virus (HCV), Ebola virus (EV), NDV and Herpes simplex virus (HSV), induce the activation of this pathway to promote entry of the virus into the host cell [68, 73–77]. Particularly, HSV makes use of an interesting strategy to enter the cell. As a consequence of the interaction of viral envelope glycoproteins with cell surface, a small amount of intracellular calcium is released, and phospholipid scramblase-1 (PLSCR1) is activated. PLSCR1 flips phosphatidylserines (PtdS) from the inner to the outer leaflet of the plasma membrane, which results in externalization of AKT. Therefore, externalized AKT interacts with viral glycoprotein B, which apparently allows AKT phosphorylation at S473 and T308 by yet undefined kinases [78, 79]. Activation of AKT pathway is required for the release of cytoplasmatic calcium that promotes the fusion of the plasma membrane and viral envelope along with the entry of viral capsids [80]. Finally, PtdS and AKT flip back to the inner leaflet of the plasma membrane in response to the interaction between PLSCR1 and viral glycoprotein L. As PtdS exposure is an apoptotic signal, HSV presumably prevents this by restoring plasma membrane architecture [79] (Supplementary Table 1).

Interestingly, some viruses co-opt host mRNA splicing machinery, either to expand their coding potential or to disrupt host mRNA splicing patterns, altering cellular mRNA processing to facilitate viral gene expression. As an example, HSV-1 protein ICP27 interacts with SR proteins, which are RNA-binding proteins essential for mRNA processing, especially splicing regulation. This interaction affects SR protein phosphorylation, resulting in the shutdown of host mRNAs splicing [81]. Additionally, the Dengue virus (DV) has been also shown to hijack U5 snRNP proteins and RBM10 to deregulate host cell splicing [82, 83]. The PI3K/AKT pathway has been widely shown to regulate mRNA processing and particularly splicing. In response to extracellular stimuli, AKT has been described to phosphorylate SR and other RNA-binding proteins such as hnRNP proteins. It has been reported that AKT can phosphorylate splicing proteins both in a direct manner and also indirectly, via SRPK1/2 [37, 84, 85]. The Human Immunodeficiency virus 1 (HIV-1) seems to promote PI3K signaling in favor of phosphorylating SR proteins in order to regulate viral mRNA splicing [37, 86]. Furthermore, the PI3K/AKT pathway plays a key role in the regulation of the HPV type 16 (HPV16) life cycle-dependent gene expression. AKT activity seems to benefit HPV16 early gene expression and suppress HPV16 late gene expression: inhibition of AKT activity leads to dephosphorylation of hnRNP L, which causes reduction in HPV16 early polyadenylation and activation of HPV16 late L1 mRNA splicing [87] (Supplementary Table 1).

The PI3K/AKT pathway can also be hijacked for viral protein expression. In the context of translation initiation, viral mRNAs can be translated in a cap-dependent or independent manner, which involves the use of internal ribosome entry sites (IRES). The AKT substrate SRSF1 (also known as SF2 or ASF) has been reported to increase the ratio between cap-dependent translation and IRES-dependent translation [88]. In particular, it has been shown that viruses like NDV and HPV16 induce PI3K/AKT/mTOR pathway activation in order to upregulate cap-dependent host machinery and facilitate viral mRNA translation [89, 90].

Regarding tumorigenesis, viral infections are associated with 10-15% of all human cancers worldwide [91]. Several viruses, including Hepatitis B virus (HBV) and HCV, Epstein-Barr virus (EBV), Kaposi’s sarcoma herpesvirus (KSHV) and HPV are oncogenic. The PI3K/AKT signaling pathway is frequently deregulated in many types of human cancers, and in particular, in virus-induced malignancies. Activation of this pathway produces an increase in cell proliferation, resistance to apoptosis, genetic instability and even changes in cytoskeletal dynamics that lead to malignant transformation of the infected cells [92, 93]. K1 and vGPCR proteins of KSHV induce AKT phosphorylation via PI3K activation, while K1 also induces PTEN inactivation. Constitutive activation of AKT enhances survival and proliferation of infected cells and has an essential role in KSHV carcinogenesis [76, 94, 95]. It has been demonstrated that LMP1 and LMP2A viral proteins are responsible for activating AKT in EBV-associated cancers [96]. HCV-infected hepatocellular carcinoma (HCC) patients show decreased levels of PTEN, which is associated with HCC pathogenesis and poor prognosis [93]. Similarly, HBx protein of HBV reduces PTEN activity and enhances carcinogenesis [97]. E6 and E7 oncoproteins of HPV, identified as those responsible for the maintenance of HPV-related oncogenesis, inhibit tumor suppressors p53 and Rb, hence preventing apoptosis. These proteins also induce the activation of the PI3K/AKT/mTOR pathway [89] and/or prevent its inactivation, as mentioned above [65] (Supplementary Table 1).

#### AKT and SARS-CoVs

Particularly, as a consequence of SARS-CoV infection, different signaling pathways are up- and downregulated. Activation of two opposing cellular programs has been demostrated: on the one hand, apoptosis to kill virus-infected cells and, on the other hand, cell survival through the production of antiviral cytokines. In particular, AKT has been shown to be a key player in the regulation of cell death and survival in cells infected by SARS-CoV [98] (Supplementary Table 1).

A pioneer study developed by Surjit and collaborators in 2004 reported that as a result of the expression of SARS-CoV N, the levels of phospho-AKT and Bcl-2 are downregulated in COS-1 monkey kidney cells, correlating with activation of caspases 3 and 7 [99]. Activation of apoptosis in these cells was independent of the p53 and Fas signaling cascade.

The same year, Morikawa and collegues showed that in confluent Vero E6 cells AKT is activated after SARS-CoV replication prior to cell death [100]. Phosphorylation in AKT S473 was detected about 8 h after infection, decreasing after 18 h. In contrast, no phosphorylation in AKT T308 was detected. In the following years, the same group reported that the AKT pathway is required for establishing persistent SARS-CoV infection in these cells and that proliferation of subconfluent Vero E6 cells ceased after SARS-CoV infection, a process accompanied by persistent AKT dephosphorylation [101, 102]. Apparently, SARS-CoV infection is made possible via the phosphorylation of AKT and JNK, both of which are induced by the viral protein N [103]. The authors concluded that four proteins, AKT, JNK, Bcl-2 and Bcl-xL are necessary for survival of persistently SARS-CoV-infected cells (Supplementary Table 1).

One year later, Chan et al. showed that the SARS-CoV M protein induces apoptosis through modulation of the AKT cell survival pathway and the release of mitochondrial cytochrome c in both HEK293T cells and transgenic *Drosophila* [104]. The same group reported 7 years later that the C-terminus of the M protein interacts with the PH domain of PDK1 and that this interaction disrupts the association between PDK1 and AKT, leading to downregulation of AKT activity [105]. This signaling cascade culminates in the activation of caspases 8 and 9.

After the COVID-19 outbreak, it was not surprising to find that AKT was among the most strongly regulated kinases following not only SARS-CoV but also SARS-CoV-2 infection [106–113].

Endocytosis of SARS-CoV-2 after binding to the ACE2 receptor occurs via the clathrin-mediated pathway, which is regulated by the PI3K/AKT signaling cascade [114]. Moreover, the reduction of ACE2 at the cell surface after SARS-CoV-2 contributes to an increase of angiotensin II in serum, which in turn can trigger the activation of the PI3K/AKT signaling pathway after binding to the angiotensin II receptor type 1 (AT1R) [114].

Consistently, a proteo-transcriptomic analysis developed by Appelberg et al. revealed that AKT signaling is modulated in Huh7 cells infected with SARS-CoV-2, showing an activation 24 h after infection. Moreover, Li et al. showed that SARS-CoV-2 infection induces autophagy and apoptosis in human microvascular endothelial and bronchial epithelial cells by inhibiting the PI3K/AKT/mTOR pathway and increasing intracellular reactive oxygen species (ROS) levels [115]. Callahan et al. demonstrated that inhibiting the AKT pathway with the inhibitor GSK690693 markedly reduced gene expression of chemokines CXCL9, CXCL10 and CXCL11 in cells infected with SARS-CoV-2, controlling hyperinflammation [116]. Ren et al. further demonstrated that SARS-CoV-2 M and N proteins can induce caspase-dependent apoptosis by physically interacting with PDK1 and preventing PDK1-AKT interaction and inhibiting AKT signaling in Vero E6 and HepG2 cells [117]. Recent work from Malgotra and Sharma revealed that activation of the AKT pathway by SARS-CoV-2 contributes to the induction of glucose uptake via glucose transporters (GLUTs), leading to increased glycolysis and viral replication in host cells [118]. Finally, Pelzl et al. also found that antibody‐mediated procoagulant platelet formation in COVID‐19 is AKT dependent, suggesting that targeting this pathway might represent a promising strategy to reduce the risk for thrombosis in patients with severe COVID-19 [119] (Supplementary Table 1).

As discussed in the following sections, these results led to the conclusion that AKT could be an interesting target in patients infected with SARS-CoV-2 and other similar viruses [120–122].

### UPR, viral infection and SARS-CoV-2

#### UPR regulation during viral infection

During the last two decades, a great deal of effort has been focused on establishing the link between ER stress, the unfolded protein response (UPR), and viral infection. Viruses induce ER stress since they require the host’s ER machinery to synthesize vast quantities of proteins so as to replicate successfully [123]. Moreover, several key stages of the viral life cycle take place in the ER, including protein synthesis and modification, genome replication and virus assembly [124].

In order to deal with ER stress and restore cellular homeostasis, the UPR is activated [125]. The three branches of the UPR function together as to decrease the load of unfolded or misfolded proteins in the ER by attenuating protein translation, expanding the ER folding capacity, increasing the ERAD, and by changing the protein secretory and intracellular trafficking routes [126]. If cells are not able to resolve ER stress during viral infection, they activate cell death pathways such as intrinsic mitochondrial apoptosis [127]. Nonetheless, several viruses are capable of hijacking the UPR to promote their survival and replication [61] (Supplementary Table 2 and Fig. 2).

Several past studies have focused on unraveling the ability of hijacking the UPR by certain viral pathogens, through the modulation of the different UPR branches. For instance, some viruses are capable of selectively activating the ATF6 branch of the UPR, inducing chaperone expression so as to synthesize viral proteins [128]. Previous research has shown that ATF6 induction is key for successful replication of the Lymphocytic Choriomeningitis virus (LCMV) [129], the African Swine Fever virus (ASFV) [130], the West Nile virus (WNV) [131], the HBV [132], and of the Zika virus (ZIKV) [133]. A recent study has also shown that ATF6 branch activation by HBx is essential for host cell survival in hepatoma cells, which could represent a key player in hepatocellular carcinoma development [134]. Moreover, the ATF6 branch was activated by the HCV core protein in a human hepatocellular carcinoma cell line. In this case, the HCV core protein induced autophagy through the activation of the ATF6 and PERK branches of the UPR (but not of the IRE1-XBP1 branch) [135]. Activation of autophagy via the induction of the ATF6 and PERK pathways was also reported in porcine cell lines infected with the Seneca Valley virus (SVV) [136] (Supplementary Table 2).

Activation of the IRE1-XBP1 branch of the UPR may also promote viral replication, since it increases the folding capacity in the ER and the synthesis of new ER membranes [137]. For instance, the exclusive activation of the IRE1-XBP1 branch leads to the replication of the IAV in human lung epithelial cells [138]. Moreover, in cells infected with the Japanese Encephalitis virus (JEV) [139, 140], the DV [140], the WNV [141] and the HBV, an activation of the IRE1-XBP1 branch is reported. For both the JEV and the DV, IRE1-XBP1 branch activation is associated with cell survival as evidenced by the fact that the knockdown of XBP1 brings along an enhanced cytopathic effect in a mice neuroblastoma cell line [140]. On the other hand, the Classical Swine Fever virus (CSFV) induces both the IRE1-XBP1 and PERK branches of the UPR, then triggering autophagy in order to enhance viral replication [142]. In addition, a recent study has revealed that Marburg virus (MARV) regulates IRE1-XBP1 in a time-dependent manner, upregulating the IRE1-XBP1 branch during the first 24 h post infection so as to promote viral replication [143]. Curiously, another study has shown that replication of Tick-borne Encephalitis flaviviruses (TBEV) in astrocytes was severely reduced when they were treated with IRE1 inhibitors before viral infection [144] (Supplementary Table 2).

Concerning the PERK branch of the UPR, ATF4 activity may play an important role in the re-establishment of cellular metabolism in virus-infected cells and in the resumption of protein synthesis. For instance, the upregulation of ATF4 has been shown to enhance the replication of HIV-1 [145]. Furthermore, both the human Cytomegalovirus (CMV) and the murine CMV induce ATF4 accumulation [146–148]. Interestingly, murine CMV prevented the expression of CHOP, a pro-apoptotic transcription factor downstream of ATF4, which could represent a viral strategy for promoting host cell survival [148]. Besides, a recent study has revealed that the Porcine Reproductive and Respiratory Syndrome virus (PRRSV) is capable of hijacking the ATF4 protein to viral replication complexes so as to promote its replication [149].

In opposition, other viruses do not induce the UPR since they have evolved strategies to suppress it. For instance, Herpes Simplex virus Type 1 (HSV-1) effectively suppresses the UPR at early stages of infection, with release of suppression at later stages as evidenced by an increased activity of eIF2α and ATF4, when virion assembly and liberation has been completed [150]. Furthermore, a recent study has revealed that the kinase activity of IRE1 was beneficial for replication of the HSV-1, while the RNase activity of IRE1 resulted pernicious in this process [151] **(**Supplementary Table 2).

#### UPR and SARS-CoVs

As described above, accumulating evidence suggests that ER stress and UPR activation are common outcomes during viral infection, and coronavirus infections are not the exception. The expression of ER-chaperones like GRP78 and GRP94 are clear indicators of ER stress. Different groups found that infection with SARS-CoV causes an increase in expression levels of GRP78 and GRP94 [152–154]. There are several aspects of coronavirus infection that may cause ER stress. Their replication occurs in the cytoplasm, strongly associated with the ER, and during this process great amounts of viral proteins are synthetized, some of which, are folded and modified in this compartment [155]. In fact, the S protein folding and maturation is mediated by ER chaperones [156]. If the accumulation of unfolded viral proteins saturates the folding capacity of the ER, this could induce ER stress and trigger the UPR. In addition, the ER-Golgi intermediate compartment (ERGIC), which is an extension of the ER, is where formation and budding of virions occur [157]. This process depletes the levels of ER membrane, which induces ER stress and activates the UPR [158, 159]. Furthermore, coronaviruses induce the formation of double membrane vesicles (DMVs) derived from the ER that also deplete membrane levels, and therefore trigger ER stress [160] (Supplementary Table 2).

In 2006, Chan et al. found that cells transfected with luciferase reporter constructs driven by GPR78 or GPR94 promoters were activated in cells infected with SARS-CoV or even merely overexpressing the S protein, thus confirming that infection with SARS-CoV induced ER stress through transcriptional activation of these chaperones [153]. Other SARS-CoV proteins such as E, M and NSP6 did not activate these promoters. S protein overexpression did not affect the activity of ATF4’s promoter, and only mildly affected CHOP´s promoter activity. When cells were co-transfected with a dominant-negative mutant of PERK or a dominant-negative mutant of eIF2α, transcription of GPR78 and GPR94 was blocked, showing that PERK activation and eIF2α phosphorylation were required for their induction. Chan et al. also found that eIF2α phosphorylation increased after infection with SARS-CoV or S protein overexpression. Finally, S protein overexpression did not significantly affect the IRE1 or ATF6 pathways. In conclusion, SARS-CoV S protein specifically activated PERK but did not affect IRE1 or ATF6. The authors suggested that this modulation of the UPR likely represents a viral strategy to combat the host’s response while facilitating viral replication, and that the effect of S was shown mainly in the transcriptional activation of chaperones, which would enhance the folding capacity of the ER. Although S activated the PERK/eif2α pathway, which blocks global protein synthesis of the cell, it had little influence over CHOP activation. CHOP activation would lead to apoptosis, which is undesirable for the virus when the infection is at an early stage. Low levels of IRE1 activation could also be beneficial for virus replication, since XBP1 upregulates chaperone expression, but high levels of activation could lead to apoptosis (Supplementary Table 2).

Three years later, Minakshi et al. showed that GRP78 and GRP94 promoters are activated when SARS-CoV 3a protein is overexpressed [161]. Of the three UPR sensors, only PERK was found to be activated in 3a expressing cells, based on the levels of eif2α phosphorylation and the inhibitory effects of a dominant negative version of eIF2α on the GRP78 promoter. 3a overexpression also increased the activity of the ATF4 and CHOP promoters. The authors concluded that this may be beneficial for viral replication, because while PERK activation leads to increased expression of ER chaperones, IRE1 and ATF6 activation would also activate ERAD, causing the degradation of viral proteins.

In 2011, DeDiego et al. found that infection with SARS-CoV lacking the E protein is attenuated in vivo [162]. To analyze the effects of the E protein, they infected cells either with SARS-CoV or a recombinant SARS-CoV lacking E protein (rSARS-CoV-ΔE) and analyzed differentially regulated genes. Most of these genes were involved in cytoplasmic, ER or mitochondrial stress, and their expression was higher in cells infected with rSARS-CoV-ΔE. In addition, levels of spliced XBP1 were higher in cells infected with rSARS-CoV-ΔE than SARS-CoV infected cells, but there was no significant activation of PERK or ATF6 pathways. These results suggest that ER stress in cells infected with SARS-CoV is downregulated when the E protein is expressed, which the authors suggest might work as a strategy for preventing premature cell death and facilitating efficient virus replication (Supplementary Table 2).

ER stress and UPR have already been associated with SARS-CoV-2 infection in previous works [163–165]. Tang´s group found in 2021 that open reading frame 8 (ORF8) protein induces ER stress and UPR: cells transfected with either of the two different genotypes of ORF8 protein (ORF8L and ORF8S) had an increase in luciferase activity of reporter constructs driven by the promoters of GRP78 or GRP94, and mRNA levels of these two chaperones were upregulated too [166]. They also observed a reduction in full-length ATF6 levels, and a 35–40% increase in cleaved ATF6 levels, indicating that the ATF6 branch of the UPR was activated. There was also an increase in XBP1 splicing, and in the amount of ERdj4 protein, a downstream target of spliced XBP1, indicating an upregulation of the IRE1 branch. However, there was no induction of the PERK branch.

As stated before, both SARS-CoV and SARS-CoV2 infection upregulate GRP78 in the infected host cells. Interestingly, Ahmed´s group suggested that GRP78, which is the main responsible for directing the misfolded proteins either for refolding or degradation, could be an interesting target for viral infections [167]. While under normal conditions GRP78 is found in the ER, it has been shown that under stress conditions this protein can translocate into the cytoplasm and on the cell membrane, where it is known as cell surface GRP78 (CS-GRP78) [168, 169]. On the other hand, there is evidence that CS-GRP78 facilitates pathogenic entry to the cell in many different types of viral infection. Moreover, bioinformatic studies suggest that CS-GRP78 can interact with the spike protein of SARS-CoV-2 and improve virus attachment and host cell entry [170]. The authors suggest that lowering the concentration of CS-GRP78 could reduce the number of internalized viral particles.

In the last months, evidence of activation of the UPR during SARS-CoV-2 infection has been rapidly accumulating. In 2021, Balakrishnan and Lai overexpressed the S protein in HEK293T cells, and found augmented ER stress (evidenced by a higher level of GRP78), a significant increase in the levels of phosphorylated eIF2α (suggesting PERK branch activation) as well as induction of autophagy and cell death [171].

That same year, Rosa-Fernandes et al. provided evidence that SARS-CoV-2 hijacks the glycosylation biosynthetic, ER-stress and UPR machineries for viral replication, using a spatio-temporal mass spectrometry-based quantitative approach comprised of proteome, membranome and N-deglycoproteome of SARS-CoV-2 infected Vero cells [172]. They also performed immunoblotting for ER-stress and UPR markers, and found increased phosphorylation levels of PERK and eIF2α, together with increased protein levels of ATF4 2 h after viral infection. They also observed higher levels of ATF6 and phosphorylated IRE1α 48 h after infection. Finally, they re-analyzed transcriptome data obtained from lung autopsies of patients who died as a result of COVID-19: differentially regulated transcripts were involved in several processes linked to ER stress, such as cell death, chaperone-mediated folding, ‘de novo’ protein folding, protein localization to ER and programmed cell death [172].

In 2021, Chuan-min´s group found that expression of SARS-CoV-2 protein ORF3a in Hela cells increased GRP78, ATF4, CHOP, cleaved ATF6 and XBP1s protein levels, indicating the induction of the three UPR signal pathways [173]. They also reported that transfection of both ATF6 and IRE1 siRNA inhibited ORF3a-induced autophagy, while transfection of PERK siRNA did not, indicating that ORF3a promotes the induction of autophagy via the ATF6 and IRE1 pathways [173].

In 2022, Galli´s group demonstrated that SARS‐CoV‐2 infection in VERO‐E6 cells stimulated the expression of the ER stress signaling protein IRE‐1α [163], and that IRE‐1α activation in SARS‐CoV‐2 infected cells is associated with the inflammatory response (NF‐kB activation and pro‐inflammatory cytokine induction), which are key pathogenic events in COVID‐19. They also show that treatment with the antiviral Nelfinavir significantly reduced IRE‐1α activity of the infected cell, and restored homeostatic processes of the host cell. They conclude UPR signaling and ER stress are main aspects of the SARS‐CoV‐2‐host interaction, apparently contributing to viral replication and to its inflammatory complications (Supplementary Table 2).

### The cross-talk between AKT and UPR pathways

**Fig. 3:**
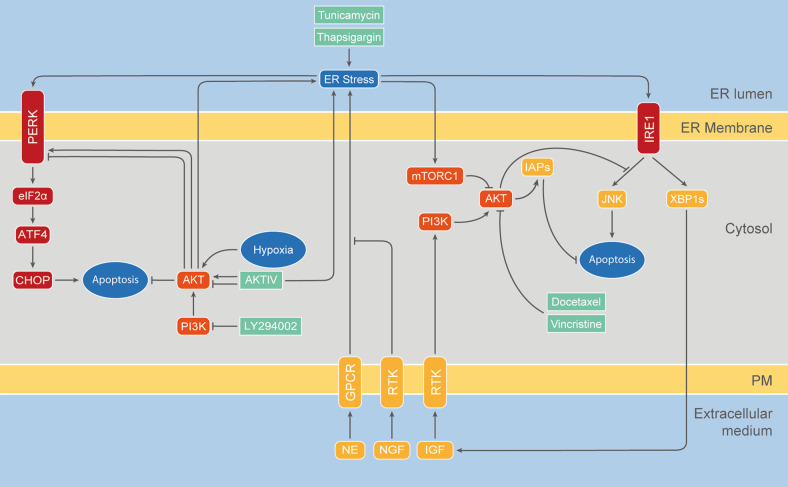
Crosstalk between the AKT and the UPR pathways. The main interactions between both signaling pathways are shown, together with the main biological processes regulated by these pathways. Pharmacological compounds targeting these feedback loops with a potential therapeutic role in SARS-CoV-2 infection are listed below.

Even though AKT and UPR pathways maintain homeostasis regulating similar fundamental biological processes, they have been studied independently from each other for a long time. It was not until 2004 that the Exton group found a link between these pathways. First, they showed that PI3K and AKT are activated in different human cancer cell lines in response to ER stressors such as thapsigargin and tunicamycin. This activation was associated with the induction of two members of the Inhibitor of Apoptosis (IAP) family of caspase suppressors, cIAP-2 and XIAP [174]. Interestingly, this work also showed that either a PI3K inhibitor or a dominant-negative AKT version reversed cIAP-2 and XIAP induction in response to ER stress and sensitized cells to cell death. Knockdown of cIAP-2 and XIAP by RNA interference was sufficient to sensitize cells to ER stress-induced death, one of the first demonstrations that activation of endogenous AKT/IAPs plays a critical role in controlling cell survival by resisting ER stress-induced cell death signaling (Fig. 3).

Two years later, in 2006, Mao et al., reported that the proapoptotic effect of norepinephrine (NE) in PC-12 cells is associated with both the activation of UPR and the inhibition of the PI3K/AKT pathway [175]. While stimulation with Nerve Growth Factor (NGF) activated PI3K/AKT and prevented ER stress-induced cell death, different PI3K inhibitors abolished this protective effect.

In 2007, Hosoi et al. discovered that PI3K/AKT is also regulated by ER stress in glial cells [33]. Interestingly, they found a dual effect: while AKT activation was upregulated by short-term exposure to ER stress, it was decreased during long-term exposure to ER stress. This was the first study to report AKT recruitment to the ER by immunohistochemistry and they also found increased AKT presence in the microsomal fraction during ER stress.

The same year, Hu et al. found that stable overexpression of XBP1s is sufficient to upregulate IGF expression and AKT phosphorylation in a zebrafish embryonic cell line (ZF4) [176]. Similarly, Zhang´s group found that XBP1 mediates the activation of AKT under ER stress in human melanoma cells [177, 178]. Remarkably, this process allowed tumor-derived cells to acquire resistance to chemotherapeutic drugs, such as docetaxel and vincristine, a phenomenon blocked by knockdown of XBP1 [177].

In 2010, Ishigaki et al. provided another piece of evidence in favor of the link between the UPR pathways and AKT [179]. Particularly, they found an induction of AKT1 during UPR, which depended on the activation of the PERK–eIF2α pathway, the upregulation of the Antagonizing Transcription Factor (AATF) and the Signal Transducer and Activator of Transcription 3 (STAT3). The activation of this signaling pathway mediated an antiapoptotic effect in rat insulinoma cells exposed to ER stress, which depended on the presence of AKT1. The same year, Qin et al., reported that ER stress-induced cell death in MEF cells is mediated by AKT inhibition, since the latter directly leads to downregulation of mTOR and enhancement of autophagy [180]. Similarly, Deldicque et al. reported that ER stress induces anabolic resistance in muscle cells in a manner that is dependent on the downregulation of AKT and consequent inhibition of mTORC1 [181]. On the other hand, Kato et al. described that mTORC1 suppressed AKT1 activity and enhanced the IRE1-JNK pathway during ER stress in the rat renal tubular epithelial cell line NRK-52E, resulting in the induction of apoptosis [182]. In summary, all these different groups have shown that the pro-apoptotic effects triggered by ER stress are alleviated by UPR-mediated activation of the AKT survival pathway, constituting an incoherent feed-forward loop.

Additionally, two different works, in 2011 and 2013, reported regulation of the UPR pathways by AKT for the first time. On the one hand, using pharmacological compounds such as the PI3K inhibitor LY294002, Mounir et al. found that AKT phosphorylates PERK at Thr799 leading to its inhibition in mammalian and *Drosophila* cells as well as in mouse mammary gland tumors [183]. On the other hand, Blaustein et al. reported that AKT phosphorylates PERK at a still unknown residue in response to hypoxia and to the antiproliferative and antiviral drug AKT-IV, leading to PERK–eIF2α activation and cell death [32]. In agreement with a positive role of AKT in PERK activation, Winnay et al. recently reported that PI3K/AKT inhibition leads to dephosphorylation of PERK at Thr980, a process associated with its inactivation [184]. The authors also observed that PI3K/AKT blockade leads to IRE1 inactivation, concluding that inhibition of the PI3K/AKT pathway blocks UPR and reduces sensitivity to ER stress-dependent apoptosis. The results of Mounir et al., Blaustein et al. and Winney et al. combined suggest that AKT might regulate the UPR by affecting PERK phosphorylation at different residues, eliciting different and even opposite responses depending on the cell context.

All together, these observations reveal the existence of a two way crosstalk between UPR and AKT pathways and suggest that they might constitute a unified homeostatic control system (Fig. 3). Feedback regulation between AKT and UPR pathways emerges as a master control mechanism of cell decision-making in terms of survival or death, showing the remarkable flexibility of signaling pathways which can direct cells to opposing fates depending on the dynamics of their activation. Recent work confirms the relevance of the crosstalk between the AKT and the UPR pathways [185–190].

### Discussion: AKT and UPR signaling as possible targets for therapies against COVID-19 and cancer

Particularly, as we have shown along this review, AKT and UPR pathways are hijacked and deregulated during virus and particularly coronavirus infection, as well as during cancer development. Cancer is one of the most common comorbidities in relation with COVID-19. Particularly, SARS-CoV-2 replication in host cells depends on altered glucose metabolism. This metabolism is similar to the Warburg effect well studied in cancer, for which AKT and UPR pathways play a key role [191, 192]. The Warburg effect appears to be involved not only in several steps of cancer progression but also of COVID-19 infection. Interestingly, Mukhopadhyay et al. highlighted the potential of AKT-related miRNAs in the development of diagnostics, biomarkers, and novel targets for SARS-CoV-2 associated with lung cancer [193]. Interestingly, two other well-known COVID-19 comorbidities, obesity and diabetes, have been linked with a cross-talk between AKT and UPR pathways [194, 195]. Therefore, the search for potential therapeutic drugs that target AKT and/or UPR pathways appears as a promising strategy in the fight against COVID-19, particularly for patients with comorbidities such as cancer, obesity and/or diabetes.

Interestingly, AKT has been recommended as a viable target to treat COVID-19 patients [114, 196, 197] (Fig. 3). On the one hand, AKT signaling has been associated with disease progression [198]. On the other hand, it has been suggested that AKT inhibition would increase the number of regulatory T cells, suppressing inflammation, promoting vascular regeneration and wound resolution [114, 120]. Consistently, it has been proposed that targeting AKT can inhibit entry and replication of SARS-CoV-2, modulate the immune response, suppress the cytokine storm, and protect against thrombosis associated with severe COVID-19 cases [119, 197, 199].

Leonardi and Proenca have also proposed a link between AKT and Fas (CD95) to quell aberrant T cell differentiation and apoptosis in COVID-19 [122]. Interestingly, Diacerein, a drug derived from anthraquinone whose active metabolite, rhein, elicits antiviral activity, inhibited the interaction between SARS-CoV protein S and ACE2, through the downregulation of AKT, among others, suggesting that rhein is a potential therapeutic agent for the treatment of SARS-CoV-2 infection [200]. Recently, green tea polyphenol catechins have been suggested as a potential therapy to treat or prevent SARS-CoV-2 infection [201]. Catechins, which display anti-inflammatory and antiviral activities, have been shown to inhibit SARS-CoV replication, potentiate adaptive immunity and attenuate acute lung injury in mice through the inhibition of the PI3K/AKT pathway [201]. Shirato et al. showed that *Asparagus officinalis* stem standardized extract (EAS) attenuates SARS-CoV-2 spike protein S1 subunit (S1)-induced IL-6 and IL-1β production by suppressing p44/42 MAPK and AKT signaling in murine primary macrophages, suggesting that EAS may be beneficial in controlling excessive inflammation in COVID-19 patients [202]. Moreover, Yang et al. have confirmed the relationship between AKT and pulmonary fibrosis caused by COVID-19, showing that D-limonene has a potential therapeutic value in SARS-CoV-2-triggered pulmonary fibrosis by inhibiting the AKT pathway [121]. Recently, Wang et al also discovered that high-cannabidiol cannabis extracts attenuated ACE2 and transmembrane protease serine 2 (TMPRSS2) expression and the induction of inflammatory proteins COX2, IL-6, and IL-8 through the AKT pathway [203]. Finally, hesperetin, a molecule with antioxidant, anti-inflammatory, and antiviral properties which is mainly found in citrus honey, has recently been suggested by Khezri et al. as a therapeutic treatment against SARS-CoV-2 infection in a probable connection with the PI3K/AKT pathway [204].

Remarkably, an antiviral drug screen performed by García Jr. et al. identified different AKT inhibitors (dactolisib, AZD2014 and torin2) as potent blockers of SARS-CoV-2 replication [205]. Moreover, Sun et al. found an AKT inhibitor, capivasertib, which restricted the entry of SARS-CoV-2 into cells under non-cytotoxic concentrations [206]. Consistently, work by Stukalov et al. found that the AKT inhibitor ipatasertib exhibited high antiviral activity against SARS-CoV-2 [111]. Finally, inhibition of AKT with the pharmacological compound MK-2206 showed a significant reduction in virus production as well [207].

Similarly, the UPR has also been recently proposed as a therapeutic target for COVID-19 (Fig. 3). As it has been pointed out, replication of coronaviruses induces UPR in the infected cells. Interfering with this response may provide new therapeutic targets and antiviral agents against COVID-19 [173, 208]. It is also known that dysregulation of the vascular barrier function contributes to the irreversible outcomes of SARS. Since the UPR modulates lung endothelial permeability, it has been suggested that UPR manipulation by pharmacologic intervention can serve to oppose the devastating outcomes of COVID-19 [209]. The fact that COVID-19 tends to affect men more severely than women has also been suggested to have a link with the UPR: estrogen production would serve to alleviate ER stress shielding women from COVID-19 complications [210]. Consistently, different authors have reported the potential utility of drugs, such as Gene-Eden-VIR/Novirin, targeting the UPR as COVID-19 therapeutic treatments [211–213]. Interestingly, Ahmed´s group suggests that GRP78, which is the main responsible for directing the misfolded proteins either for refolding or degradation, could be an interesting target for both viral infections and cancer [167]. CS-GRP78 is found in many types of aggressive cancers. In non-small-cell lung cancer (NSCLC) and glioblastoma multiforme (GBM), for example, overexpression of CS-GRP78 is one of the responsibles for radio-resistance [214], while administration of anti-GRP78 delayed tumor growth and enhanced the efficacy of the radiation treatment. Other groups also found that targeting CS-GRP78 improves treatment efficacy [215, 216]. Moreover, crosstalk between GRP78 and AKT has been reported several times [217]. Reducing the concentration of CS-GRP78 could both reduce the number of internalized viral particles and the cancer-associated resistance, making this protein an attractive target for patients with cancer, COVID-19, or both [170]. Finally, autophagy, a process strongly regulated both by AKT and UPR pathways, as it has been described above, has been also suggested as a potential target for anti-COVID-19 therapies [218, 219].

In a context in which the use of antitumor therapies such as chemotherapy or radiotherapy generate immunosuppression [7, 8], the search for cancer therapies that do not compromise patients immunologically is urgent. Drug repositioning, the investigation of existing drugs for new therapeutic purposes, could be a therapeutic alternative for cancer and/or COVID-19 patients [11, 205, 206]. Drug repositioning could significantly reduce time and costs compared to de novo drug discovery. For instance, Nelfinavir, an antiretroviral drug used to treat HIV, has been shown to regulate AKT as well as the UPR and has been recently proposed both in cancer and in COVID-19 therapy [220–222]. Similarly, the broad spectrum corticosteroid Dexamethasone has been described to modify both, AKT as well as UPR activities and has been used in cancer and COVID-19 therapy [223–225].

On the other hand, pharmacological treatment can complement the use of vaccines, taking into account the challenges in production, affordability, allocation and global access to them [226]. Moreover, the efficacy of vaccines, designed on the basis of specific viral nucleotide or protein sequences, is challenged by the emergence of new SARS-CoV-2 variants, due to its high mutation rate [227]. The fact that some people, especially elderly or those with comorbidities such as cancer, are susceptible to severe manifestations of COVID-19 or even to death despite a complete vaccination schedule, makes the search for drugs to complement vaccination essential [228]. In other cases, such as immunosuppressed patients who cannot develop an immunological response to vaccines, this need is even more evident. In this sense, searching for pharmacological therapies associated with human proteins, with a lower mutation rate than viral proteins, could be an effective and complementary strategy to the use of vaccines. Furthermore, it could be relevant in the potential case of future epidemics or pandemics.

## Supplementary information

Supplementary tables legends

Author contribution form

Supplementary Table 1

Supplementary Table 2

## Data Availability

All data are included in this article and its supplementary materials.
