# Supplementary material for: Live and let die: signaling AKTivation and UPRegulation dynamics in SARS-CoVs infection and cancer
Source: Cell Death Dis. 2022 Oct 3;13(10):846. doi: 10.1038/s41419-022-05250-5 (PMC9529164; doi:10.1038/s41419-022-05250-5)
Supplement: Supplementary file 1 — Supplementary tables legends [file 41419_2022_5250_MOESM1_ESM.docx]

###### **Supplementary information**

###### **Table Legends**

**Supplementary Table 1. AKT and viral infection**

(a) Examples of different viruses that exploit and co-opt the AKT pathway and their mechanisms of action. (b) Hijacking of the AKT pathway by SARS-CoVs.

**Supplementary Table 2. UPR and viral infection**

(a) Examples of different viruses that exploit and co-opt the UPR pathways and their mechanisms of action. (b) Hijacking of UPR pathways by SARS-CoVs.
