# Supplementary material for: Live and let die: signaling AKTivation and UPRegulation dynamics in SARS-CoVs infection and cancer
Source: Cell Death Dis. 2022 Oct 3;13(10):846. doi: 10.1038/s41419-022-05250-5 (PMC9529164; doi:10.1038/s41419-022-05250-5)
Supplement: Supplementary file 2 — Author contribution form [file 41419_2022_5250_MOESM2_ESM.pdf]

DECLARATION OF CONTRIBUTIONS TO ARTICLE

ADMC

Manuscript Number: CDDIS-22-2564R

Journal Name: *Cell Death & Disease* (the 'Journal')

Proposed Title of the Contribution:

Live and let die: Signaling AKTivation and UPRegulation dynamics in SARS-CoVs infection and cancer (the 'Contribution)

Author(s):

Suaya, Mariana*; Sánchez, Gonzalo Manuel*; Vila, Antonella*; Amante, Analía*; Cotarelo, María; García Carrillo, Mercedes* and Blaustein, Matías (the 'Authors')

For all *CDDis* articles, each person named as an author in the published version must be able to show he or she has contributed substantially to the article.

Authorship credit should be based on 1) substantial contributions to conception and design, acquisition of data, or analysis and interpretation of data; 2) drafting the article or revising it critically for important intellectual content; and 3) final approval of the version to be published. Authors should meet conditions 1, 2 and 3.

Any person who cannot be shown to have made a substantial contribution to the article cannot be listed as an author in the final version. The name of any person who is deemed to have made a minor contribution can, however, appear in the Acknowledgments section of the article.

Please complete the table below to indicate the contributions of all named authors to the manuscript.

Author Full Name:	Specification of Contribution to the Manuscript:
Mariana Suaya	Designed the supplementary tables and wrote the manuscript.
Gonzalo Manuel Sánchez	Designed the figures and wrote the manuscript.
Antonella Vila	Designed the supplementary tables and wrote the manuscript.
Analía Amante	Designed the supplementary tables and wrote the manuscript.
María Cotarelo	Revised and edited the manuscript.
Mercedes García Carrillo	Designed the supplementary tables and wrote the manuscript.
Matías Blaustein	Conceptualized, designed and wrote the manuscript.

Please complete the table below to indicate the contributions of all named authors to the figures.

Figure 1:

Gonzalo Manuel Sánchez designed the figure.
Karin E. Giacomuzzi edited the final version.
All contributing authors revised the figure.

Figure 2:

Gonzalo Manuel Sánchez designed the figure.
Karin E. Giacomuzzi edited the final version.
All contributing authors revised the figure.

Figure 3:

Gonzalo Manuel Sánchez designed the figure.
Karin E. Giacomuzzi edited the final version.
All contributing authors revised the figure.

Figure 4:

Figure 5:

Figure 6:

Signed for and on behalf of the Author(s):	Print Name:	Date:
	Matías Blaustein	September 6th, 2022
