# Supplementary material for: Live and let die: signaling AKTivation and UPRegulation dynamics in SARS-CoVs infection and cancer
Source: Cell Death Dis. 2022 Oct 3;13(10):846. doi: 10.1038/s41419-022-05250-5 (PMC9529164; doi:10.1038/s41419-022-05250-5)
Supplement: Supplementary file 3 — Supplementary Table 1 [file 41419_2022_5250_MOESM3_ESM.docx]

**A**

| **Virus** | **AKT pathway protein** | **Viral protein** | **AKT pathway Regulation** | **Biological process** | **Reference** |
| --- | --- | --- | --- | --- | --- |
| **Influenza A Virus**  **(IAV)** | PI3K | NS1 | Activation | Virus entry  Host cell survival | Ehrhardt *et al.* (2006)  Li *et al.* (2008) |
|  | AKT | NS1 | Activation | Host cell survival | [Matsuda *et al.*](https://www.zotero.org/google-docs/?fdapZz) (2010)  Ehrhardt *et al.* (2006) |
| **Simian virus 40 (SV40)** | PP2A | ST | Activation | Host cell survival | Yuan *et al.* (2002)  [Dunn and Connor](https://www.zotero.org/google-docs/?fdapZz) (2012) |
| **Human papillomavirus (HPV)** | PP2A | E7 | Activation | Host cell survival | Pim *et al.* (2005) |
|  | PI3K | E6 and E7 | Activation | Host cell survival  Suppress apoptosis of the infected cell | Zhang *et al.* (2015) |
|  | AKT | hnRNP L | Activation | Regulation of viral mRNA processing | Kajitani *et al.* (2017) |
|  | AKT | E6 | Activation | Increase in viral protein synthesis | Zhang *et al*. (2015) |
| **Newcastle Disease virus (NDV)** | PI3K | - | Activation | Virus entry  Host cell survival | Kang *et al.* (2017)  Blanco *et al.* (2020) |
|  | mTOR | NP | Activation | Increase in viral protein synthesis | Zhan *et al*. (2020) |
| **Respiratory Syncytial virus (RSV)** | PI3K | NS1 and NS2 | Activation | Host cell survival | Bitko *et al.* (2007)  Groskreutz *et al.* (2007) |
| **Sendai virus (SV)** | PI3K | - | Activation | Host cell survival | Blanco *et al.* (2020)  White *et al.* (2011) |
| **Vaccinia virus (VACV)**  **and Cowpox virus (CPXV)** | PI3K | - | Activation | Host cell survival  Viral replication | [Dunn and Connor](https://www.zotero.org/google-docs/?fdapZz) (2012)  Soares *et al*. (2009) |
| **Vaccinia virus (VACV)** | PI3K | - | Activation | Regulation of viral morphogenesis | [Dunn and Connor](https://www.zotero.org/google-docs/?fdapZz) (2012)  Diehl and Schaal (2013)  McNulty *et al.* (2010) |
| **Herpes Simplex virus (HSV)** | PI3K | - | Activation | Virus entry, filopodia formation and cell-to-cell fusion | Tiwari and Shukla (2010) |
|  | AKT | Glycoprotein B | Activation | Virus entry | Cheshenko *et al.* (2013, 2014, 2018) |
| **Hepatitis B virus (HBV)** | PTEN | HBx | Activation | Host cell survival  Carcinogenesis | Chung *et al.* (2003) |
| **Hepatitis C virus (HCV)** | PI3K | E2 | Activation | Virus entry | Liu *et al.* (2012) |
|  | PTEN | NS5A | Activation | Host cell survival | Cheng *et al.* (2015) |
| **Ebola virus (EV)** | PI3K | - | Activation | Virus entry | Saeed *et al.* (2008) |
| **Human Immunodeficiency virus 1 (HIV-1)** | PI3K | - | Activation | Regulation of viral mRNA splicing | [Dunn and Connor](https://www.zotero.org/google-docs/?fdapZz) (2012)  Hillebrand *et al.* (2014) |
| **Kaposi’s sarcoma herpesvirus (KSHV)** | PI3K | K1 and vGPCR | Activation | Host cell survival and proliferation  Carcinogenesis | Liu and Cohen (2015)  Sodhi *et al.* (2004)  Tomlinson and Damania (2004) |
|  | PTEN | K1 | Activation | Host cell survival and proliferation  Carcinogenesis | Liu and Cohen (2015)  Tomlinson and Damania (2004) |
| **Epstein-Barr virus (EBV)** | PI3K | LMP1 and LMP2A | Activation | Host cell survival and proliferation  Carcinogenesis | Chen (2012) |

**B**

| **Virus** | **PI3K/AKT pathway protein** | **Viral protein** | **AKT pathway Regulation** | **Biological process** | **Reference** |
| --- | --- | --- | --- | --- | --- |
| **SARS-CoV-1** | AKT | N | Inhibition | Apoptosis | Surjit *et al.* (2004) |
| **SARS-CoV-1** | AKT | N | Activation | Persistent viral infection | [Mizutani](https://www.zotero.org/google-docs/?fdapZz) *et al.* (2004, 2005, 2006) |
| **SARS-CoV-1** | PDK1 | M | Inhibition | Apoptosis | Tsoi *et al.* (2014) |
| **SARS-CoV-2** | AKT | S | Inhibition | Autophagy  Apoptosis | Li *et al*. (2021) |
| **SARS-CoV-2** | AKT | S | Activation | Inflammation | [Shirato](https://pubmed.ncbi.nlm.nih.gov/?term=Shirato+K&cauthor_id=34684771) *et al.* (2021) |
| **SARS-CoV-2** | AKT | - | Activation | Increased glycolysis Viral replication | Malgotra and Sharma (2021) |
| **SARS-CoV-2** | PIK3AP1 | - | Upregulation | Inflammation | Callahan *et al*. (2021) |
| **SARS-CoV-2** | PDK1 | M/N | Inhibition | Apoptosis | [Ren](https://pubmed.ncbi.nlm.nih.gov/?term=Ren+Y&cauthor_id=34513728) *et al.* (2021) |
| **SARS-CoV-2** | AKT | - | Activation | Procoagulant platelet formation  Thrombosis | Pelzl *et al.* (2021) |
