# Supplementary material for: Live and let die: signaling AKTivation and UPRegulation dynamics in SARS-CoVs infection and cancer
Source: Cell Death Dis. 2022 Oct 3;13(10):846. doi: 10.1038/s41419-022-05250-5 (PMC9529164; doi:10.1038/s41419-022-05250-5)
Supplement: Supplementary file 4 — Supplementary Table 2 [file 41419_2022_5250_MOESM4_ESM.docx]

**A**

| **Virus** | **UPR protein** | **Viral protein** | **UPR regulation** | **Biological process** | **Reference** |
| --- | --- | --- | --- | --- | --- |
| **Lymphocytic Choriomeningitis Virus (LCMV)** | ATF6 | GPC (Viral Glycoprotein Precursor) | Activation | Viral replication  Host cell survival | Pasqual *et al.* (2011) |
| **African Swine Fever Virus (ASFV)** | ATF6 | - | Activation | Viral replication | Galindo *et al.* (2012) |
| **Zika Virus (ZIKV)** | ATF6 | - | Activation | Viral replication | Tan *et al.* (2018) |
| **Hepatitis C Virus (HCV)** | ATF6 | HCV core protein | Activation | Autophagy | Wang *et al.* (2014) |
| **Seneca Valley Virus (SVV)** | ATF6 | - | Activation | Autophagy  Viral replication | Hou *et al.* (2019) |
| **Influenza A Virus**  **(IAV)** | IRE1 | - | Activation | Viral replication | Hassan *et al.* (2012) |
| **Japanese Encephalitis Virus (JEV)** | IRE1 | prM, E, NS1, NS2A, NS2B and NS4B | Activation | Cell survival | Yu *et al.* (2006) |
| **Dengue Virus serotype 2 (DENV-2)** | IRE1 | NS2B-3 (NS2B and NS3 fusion) | Activation | Cell survival | Yu *et al.* (2006) |
| **West Nile Virus (WNV)** | ATF6 and  IRE1 | - | Activation | Viral replication  Host cell survival | Ambrose *et al.* (2013)  Medigeshi *et al.* (2007) |
| **Hepatitis B Virus (HBV)** | ATF6 and  IRE1  PERK | HBx | Activation  Inhibition | Viral replication  Host cell survival | Li *et al.* (2007, 2017)  Li *et al*. (2017) |
| **Classical Swine Fever Virus (CSFV)** | IRE1 | - | Activation | Autophagy  Viral replication | Zhu *et al.* (2021) |
| **Marburg Virus (MARV)** | IRE1 | GP and VP30 | Activation | Viral replication | Rohde *et al.* (2019) |
| **Tick-borne Encephalitis Virus (TBEV)** | IRE1 | - | Activation | Viral replication | Breitkopf *et al.* (2021) |
| **Langat Virus (LGTV)** | IRE1 | - | Activation | Viral replication | Breitkopf *et al.* (2021) |
| **Human Immunodeficiency virus 1 (HIV-1)** | PERK | - | Activation | Viral replication | Caselli *et al.* (2012) |
| **Human Cytomegalovirus (HCMV)** | PERK | pUL38 | Activation | Viral replication  Host cell survival | Isler *et al.* (2005)  Xuan *et al.* (2009) |
| **Murine Cytomegalovirus (MCMV)** | PERK | - | Activation | Viral replication  Host cell survival | Qian *et al.* (2012) |
| **Porcine Reproductive and Respiratory Syndrome Virus (PRRSV)** | PERK | NSP2 and NSP3 | Activation | Viral replication | Gao *et al.* (2019) |

**B**

| **Virus** | **UPR protein** | **Viral protein** | **UPR regulation** | **Biological process** | **Reference** |
| --- | --- | --- | --- | --- | --- |
| **SARS-CoV-1** | PERK | Whole virus  S | Activation | Viral replication  Host cell survival | [Chan *et al.*](https://www.zotero.org/google-docs/?fdapZz) (2006)  [Yeung *et al.*](https://www.zotero.org/google-docs/?fdapZz) (2008)  [Tang *et al.*](https://www.zotero.org/google-docs/?fdapZz) (2017) |
| **SARS-CoV-1** | PERK | 3a | Activation | Viral replication  Host cell survival | [Minakshi *et al.*](https://www.zotero.org/google-docs/?fdapZz) (2009) |
| **SARS-CoV-1** | IRE1 | E | Inhibition | Viral replication  Host cell survival | [DeDiego *et al.*](https://www.zotero.org/google-docs/?fdapZz) (2011) |
| **SARS-CoV-2** | PERK, ATF6 and IRE1 | ORF8 | Activation | Viral replication  Host cell survival | [Rashid *et al.*](https://www.zotero.org/google-docs/?fdapZz) (2021) |
| **SARS-CoV-2** | CS-GRP78 | S | Activation | Viral entry | [Ibrahim *et al.*](https://www.zotero.org/google-docs/?fdapZz) (2020) |
| **SARS-CoV-2** | PERK | S | Activation | Viral replication  Host cell survival | Balakrishnan and Lai (2021) |
| **SARS-CoV-2** | PERK, ATF6 and IRE1 | Whole virus | Activation | Viral replication  Host cell survival | Rosa-Fernandes *et al.* (2021) |
| **SARS-CoV-2** | PERK, ATF6 and IRE1 | ORF3a | Activation | Viral replication  Host cell survival | Su *et al.* (2021) |
| **SARS-CoV-2** | IRE1 | Whole virus | Activation | Viral replication  Host cell survival | [Bartolini *et al.*](https://www.zotero.org/google-docs/?fdapZz)  (2022) |
